# Follicular Skin Disorders, Inflammatory Bowel Disease, and the Microbiome: A Systematic Review

**DOI:** 10.3390/ijms251810203

**Published:** 2024-09-23

**Authors:** Lauren Fleshner, Katie Roster, Banu Farabi, Rahim Hirani, Katharine Tepper, Capecomorin S Pitchumoni, Bijan Safai, Shoshana Marmon

**Affiliations:** 1 School of Medicine, New York Medical College, Valhalla, NY 10595, USA; lfleshne@student.nymc.edu (L.F.);; 2 Dermatology Department, Georgetown University School of Medicine, Medstar Washington Hospital Center, Washington, DC 20007, USA; 3 Dermatology Department, NYC Health + Hospital/Metropolitan, New York, NY 10029, USA; 4 Department of Medicine, Saint Peter’s University Hospital, New Brunswick, NJ 08901, USA; 5 Department of Medicine, NYC Health + Hospital/South Brooklyn Health, Brooklyn, NY 11235, USA

**Keywords:** human microbiome, inflammatory bowel disease, hidradenitis suppurativa, immune system, skin disease, gut–skin axis, gut flora, dysbiosis, follicular disorders, systematic review

## Abstract

Follicular skin disorders, including hidradenitis suppurativa (HS), frequently coexist with systemic autoinflammatory diseases, such as inflammatory bowel disease (IBD) and its subtypes, Crohn’s disease and ulcerative colitis. Previous studies suggest that dysbiosis of the human gut microbiome may serve as a pathogenic link between HS and IBD. However, the role of the microbiome (gut, skin, and blood) in the context of IBD and various follicular disorders remains underexplored. Here, we performed a systematic review to investigate the relationship between follicular skin disorders, IBD, and the microbiome. Of the sixteen included studies, four evaluated the impact of diet on the microbiome in HS patients, highlighting a possible link between gut dysbiosis and yeast-exclusion diets. Ten studies explored bacterial colonization and HS severity with specific gut and skin microbiota, including Enterococcus and Veillonella. Two studies reported on immunological or serological biomarkers in HS patients with autoinflammatory disease, including IBD, and identified common markers including elevated cytokines and T-lymphocytes. Six studies investigated HS and IBD patients concurrently. Our systematic literature review highlights the complex interplay between the human microbiome, IBD, and follicular disorders with a particular focus on HS. The results indicate that dietary modifications hold promise as a therapeutic intervention to mitigate the burden of HS and IBD. Microbiota analyses and the identification of key serological biomarkers are crucial for a deeper understanding of the impact of dysbiosis in these conditions. Future research is needed to more thoroughly delineate the causal versus associative roles of dysbiosis in patients with both follicular disorders and IBD.

## 1. Introduction

Follicular skin disorders (FSDs) are a group of heterogeneous, inflammatory conditions that occlude the hair follicle, typically manifesting as painful skin eruptions. These include pilonidal cysts, acne conglobata, dissecting cellulitis of the scalp, folliculitis decalvans, and perifolliculitis capitis abscedens et suffodiens. Hidradenitis suppurativa (HS) is characterized by deep-seated nodules and abscesses that are often debilitating to patients, leading to a significant decline in quality of life (Goldburg, [1]). While the exact etiology of HS is not well established, it is believed to be associated with dysregulated inflammatory cytokine response and/or an altered human microbiome [1,2]. The estimated prevalence of HS is approximately 0.0003–4.10%, and it is more commonly diagnosed in women and African Americans [1]. HS is a clinical diagnosis that can manifest in various ways; the most commonly utilized HS classification system is the Hurley staging system, which scores patients based on disease severity [1].

HS has been associated with systemic autoinflammatory disorders, such as inflammatory bowel disease (IBD), including Crohn’s disease (CD) and ulcerative colitis (UC) in multiple studies [2,3]. Furthermore, HS and IBD share common clinical manifestations, genetic susceptibility, and immunologic features (Chen, [2]). While the pathophysiology linking HS and IBD is unclear, studies have suggested that IBD and its subtypes may have a causal effect on the development of HS [4,5]. Previous studies have identified the coexistence of HS and IBD, particularly among women and Black individuals [6,7]. Given the high comorbidity, screening for IBD among patients with HS is recommended by current United States and Canadian HS Foundation Screening Guidelines (Garg [8]).

Emerging evidence suggests that both the gut and skin microbiota play a key role in pathogenicity, severity, and management of inflammatory diseases [9,10]. Specifically, the gut and skin microbiota may be responsible for triggering some cutaneous manifestations of autoinflammatory systemic diseases, such as IBD [11]. Many studies have also linked gut and skin dysbiosis to the development and increasing burden of both HS and IBD [9,12].

While dysbiosis of the gut microbiome has demonstrated involvement in the pathogenesis of both IBD and HS [2,12,13], the impact of the skin, oral and blood microbiota, on HS and FSDs remains poorly studied. Here, we systematically reviewed the literature to examine the relationship of the human microbiome with IBD and FSDs.

## 2. Methods

In accordance with the Preferred Reporting Items for Systematic Reviews and Meta-analyses (PRISMA) guidelines, a systematic review was conducted across five major databases for pertinent literature: PubMed, EMBASE, Web of Science, Cochrane Library, and Google Scholar (Page, [14]). The databases were searched from their respective inception until 2024. Covidence (Melbourne, Australia) was utilized as a central online systematic review manager. Peer-reviewed, original research involving any follicular skin disorder (HS/acne inversa, pilonidal sinus, acne conglobata, dissecting cellulitis of the scalp, folliculitis decalvans, perifolliculitis capitis abscendens et suffodiens), IBD (including Crohn’s disease, ulcerative colitis, indeterminate colitis) and the microbiome (oral, gastrointestinal, or skin) was included. Studies looking at dietary factors/antibiotics on modulating microbiomes were also included. Only empirical studies evaluating human subjects were considered. Studies were excluded if they were not in English, were “gray literature” (abstracts, posters, case reports) or non-original research (systematic reviews, meta-analyses). Studies evaluating pediatric patients or pregnant populations were excluded from analysis as their microbiomes may differ from other populations. The full search strategy can be referenced in the Supplementary Data. Two authors independently reviewed abstracts for inclusion, and disagreements were resolved by a third reviewer. Full texts were subsequently reviewed and included if they met the eligibility criteria. Data extraction from full text articles were independently collected from two authors to minimize reporting bias and human error. Any discrepancies were reviewed and resolved by a third author.

Studies included for analysis were aggregated into three categories evaluating the gut–skin axis: impact of diet, colonization of the skin and/or gut, or immunological/serological biomarkers (Table 1). Quality assessments (Table 2) were also conducted using the Newcastle–Ottawa Scale, which is a rating system evaluating cohort selection, comparability, and outcome measures. Assessments were completed by two independent reviewers, and any disagreements were resolved by discussion. This review has been registered with the Open Science Framework https://doi.org/10.17605/OSF.IO/8V2JE.

## 3. Results

**Figure 1:**
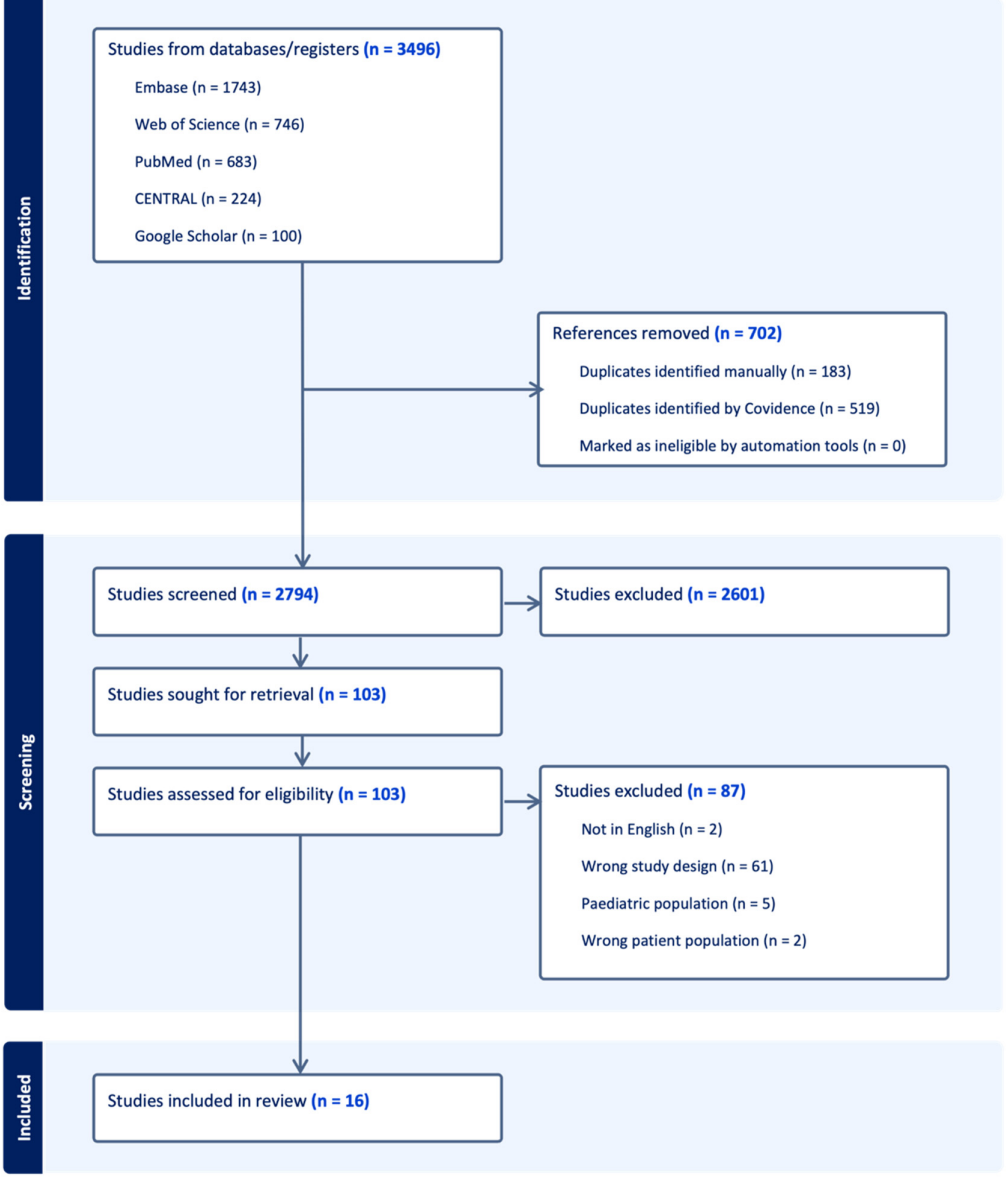
PRISMA diagram depicting selection criteria for inclusion. Generated by Covidence.

The database search yielded 2794 studies, which were initially screened by title and abstract for relevance. Among these, 103 studies underwent full-text review. Ultimately, 16 studies were included in our review. The PRISMA diagram detailing eligibility criteria is outlined in Figure 1. All of the studies included for analysis and their characteristics are described in Table 1.

### 3.1. Dietary Effects on the Microbiome

Diet has been increasingly recognized as a factor in shaping the gut microbiome and influencing inflammatory diseases. Four studies examined how dietary interventions may impact the gut microbiome in patients with inflammatory bowel disease and the development or severity of HS (Table 2) [15,17,18,19]. Aboud et al. studied HS patients, including 5% with comorbid Crohn’s disease, and found that 70% of those who excluded yeast from their diet experienced an improvement in HS symptoms, with 87% having immediate recurrences of their disease within a week of reintroducing yeast into the diet (Aboud [15]). Furthermore, this study reported that both CD and HS have been linked to anti-Saccharomyces cerevisiae antibodies (IgG family), connecting HS, CD, and food intolerances to gut dysbiosis (Aboud [15]). Similarly, a large multicentric study found that anti-Saccharomyces cerevisiae antibodies were more frequent in patients with severe HS compared to those with psoriasis vulgaris and healthy controls, highlighting the potential association between anti-Saccharomyces cerevisiae antibodies, gut dysbiosis, and systemic inflammation in HS. The presence of these antibodies against yeast in both CD and HS suggests that gut dysbiosis may lead to immune responses to certain foods, implying that specific diets could exacerbate symptoms by aggravating gut dysbiosis and food intolerances, linking them to disease progression (Assan [19]).

In another study, 20 patients with HS were given a brewer’s yeast exclusion diet, which involves avoiding foods and products containing brewer’s yeast, which is a type of yeast used in beer production and baking. After three months, there were significant reductions in pain (*p* = 0.006), the number of impaired days (*p* = 0.007), inflammation intensity (*p* = 0.018), and discharge intensity (*p* = 0.005) compared to baseline (Colboc [18]). Similarly, Cannistrà et al. studied twelve patients with HS who began following a brewer’s yeast-free diet for 12 months, leading to the stabilization and regression of skin lesions, with recurrence occurring upon reintroducing brewer’s yeast or wheat. Patients on this elimination diet reported improved quality of life, including improved sexual activity (Cannistrà [17]).

### 3.2. Bacteria Colonization

Bacterial colonization shaped immune responses and inflammatory pathways associated with various skin and systemic diseases, including HS. Research has increasingly focused on understanding the specific bacterial species involved in HS and how alterations in the skin and gut microbiome may influence disease severity and progression. Ten studies evaluated species of bacteria colonizing either the skin or gut microbiome. Of these, nine evaluated HS and one evaluated folliculitis decalvans [16,20,21,22,23,24,25,26,27,28]. Table 3 illustrates studied microbiota, their compositional changes, and potential mechanisms.

Cronin et al. found that 40% of patients with HS had a fecal microbiota configuration similar to patients with CD, including the presence of *Enterococcus, Veillonella, and Escherichia shigella* species (Cronin [16]). Furthermore, McCarthy et al. reported that Ruminococcus gnavus, a bacterium found to be more abundant in patients with Crohn’s disease, was also found to be more abundant in individuals with HS (McCarthy [21]). Among patients with folliculitis decalvans, an unbalanced subepidermal microbiota, compared to healthy control subjects, while evaluating *Staphylococcus aureus* on lesioned skin was found, potentially providing a reservoir for abnormal flora, as clinical improvement upon treatment was associated with a disappearance of *S. aureus*. (Matard [25]). Similarly, among the entire cohort of patients with HS, *S. auerus* grew on skin folds (Marzano [28]).

Hsu et al. analyzed DNA from 10 healthy control (HC) samples, 17 pre-treatment with Adalimumab HS samples, and 7 post-treatment HS samples. There was a dominance of *Prevotella* spp. (mean difference: −11.7%, 95% Cl = −18.2 to −5.3%, q = 0.033) and *Peptoniphilus* spp. (mean difference: −10.4%, 95% Cl = −14.9 to −5.9%, q = 0.004) at HS lesional sites. *Paucibacter* spp. *and Caulobacter* spp. were significantly more abundant in HCs with *Paucibacter* sxpp. showing a mean difference of 31.1% (95% Cl = 4.9–57.2%, q = 0.001) and *Caulobacter* spp. showing a mean difference of 2.4% (95% Cl = 0.6–4.3%, q = 0.004). No significant changes in the microbiome were observed during adalimumab treatment, suggesting that dysbiosis in severe HS may be due to permanent skin damage that does not recover even after treatment. TNF-α inhibitors, like adalimumab, target inflammation but may not significantly affect the skin microbiome, possibly explaining the high recurrence rate after stopping treatment (Hsu [27]).

Lam et al. compared the bacterial communities in the gut and skin of HS patients and healthy controls, finding no significant differences in bacterial richness (*p* = 0.483) or in other metrics that assess bacterial diversity and abundance, such as the Shannon index (*p* = 0.821), inverse Simpson index (*p* = 0.916), used to identify bacterial richness, or community structure based on Bray–Curtis (*p* = 0.106), to quantify dissimilarity among species composition, and Jaccard (*p* = 0.103) metrics, to gauge similarity and diversity among present colonies. The fecal microbiome of both groups was predominantly composed of *Firmicutes* (55.3% in HS, 56.0% in controls) and *Bacteroides* (15.6% in HS, 14.9% in controls). LEfSe (Linear Discriminant Analysis Effect Size), a statistical method used to identify bacterial species that differ significantly between two or more groups, revealed significant taxonomic differences, including the presence of *Robinsoniella* and *Sellimonas* in HS patients but not in controls (Lam [10]). This contrasts with Öğüt et al., who reported a significantly reduced diversity in HS patients (Shannon index *p* = 0.048), and a distinctive gut microbiome Bray-Curtis (*p* = 0.01), and Jaccard (*p* = 0.007), compared to healthy controls (Öğüt [23]).

Nikolakis et al. evaluated the bacterial colonization in HS and its correlation to Hurley stage; a staging is a system used to classify the severity of HS ranging from mild isolated lesions (Stage I) to severe (Stage III). They reported that the isolation of both aerobic and anaerobic bacteria independently predicted a higher severity of HS (*p* = 0.004) in a multivariate logistic regression analysis [22]. Riverain-Gillet similarly reported an increasing abundance of anaerobes (*p* = 0.00021) among HS skinfolds, particularly *Prevotella, Actinomyces, Campylobacter ureolyticus,* and *Mobiluncus* (Riverain-Gillet [26]). Additionally, Guenin-Macé et al. evaluated a dysregulation of tryptophan catabolism among HS lesions, potentially providing a microbiological and immunological link to the disease (Guenin-Macé [24]).

Interestingly, two studies reported contradicting results; Eppinga et al. found that a depletion of *Faecalibacterium*, commonly associated with IBD, was found in patients with psoriasis rather than HS, whereas Cronin et al. reported the depletion of *Faecalibacterium* to be positively associated with HS [16,20]. However, Eppinga et al. found that patients with contaminant HS and IBD had the greatest decrease in *Faecalibacterium* and an increase in *E. coli.*

### 3.3. Immunological/Serological Biomarkers

In addition to bacterial colonization, overlapping biomarkers—molecules such as proteins, cytokines, or other substances found in tissues or blood that indicate a specific disease process—between autoinflammatory diseases offer insight into the immune dysfunction underlying HS. Two studies evaluated immunological or serological biomarkers associated with autoinflammatory diseases, including HS and perianal Crohn’s disease [4,28]. Marzano et al. reported supporting evidence of common genetic and cytokine profiles with Crohn’s disease, including Fas ligand, cluster of differentiation 40 (CD40+) ligand, interleukin-8 (IL-8), chemokine (C-X-C Motif) ligands 1, 2, 3 (CXCL 1/2/3), CXCL 16, and CCL5, also known as regulated on activation, normal T-cell expressed and secreted (RANTES) [28]. Giudici et al. found that in both patients with HS and CD, significantly higher CD4+ and CD16+ T-lymphocytes counts in CD fistulas and HS lesions, supporting a potential role in the pathogenesis of both immune conditions (Giudici, [3]).

## 4. Discussion

This systematic review of the literature highlights key features of the microbiome and its role in follicular skin disorders and IBD. Specifically, our findings underscore the significant interplay between the microbiome dysbiosis, diet, and the immune system which may contribute to the development of HS and IBD. This aligns with previous systematic reviews summarizing data on HS and the microbiome, emphasizing the important association of dysbiosis in HS and IBD [9,13].

The studies exploring the impact of diet on the microbiome in patients with HS suggest that diets excluding yeast containing Saccharomyces cerevisiae may be beneficial for patients with HS [15,17,18]. The impact of diet on HS, particularly the exclusion of yeast-containing foods, was consistent across multiple studies [15,17,18,19]. This link between dietary yeast and HS symptomatology may be mediated through gut dysbiosis and immune responses involving anti-*Saccharomyces cerevisiae* antibodies. These antibodies may indicate an abnormal immune response to certain foods. Together, these studies highlight the importance of dietary considerations in managing HS symptoms, suggesting that individualized dietary plans could be an adjunctive treatment strategy to improve patient outcomes.

Our review also revealed significant alterations in the microbiome composition of HS patients, including the presence of *Prevotella* spp., *Peptoniphilus* spp., *Enterococcus*, *Veillonella*, and *Escherichia shigella* spp. [16,27]. These bacterial species are associated with inflammatory conditions, and their presence may exacerbate the immune response in HS. Moreover, the similarities in fecal microbiota between patients with HS and those with IBD suggest a common underlying inflammatory pathway between these conditions. Both HS and Crohn’s disease have shared genetic and cytokine profiles, including the involvement of cytokines such as IL-1β and TNF-α, indicating that HS and Crohn’s disease may share therapeutic targets. Further research into these common pathways may help with the development of novel therapies.

One limitation of our study is the lack of data on FSDs other than HS. Of the 16 studies included for analyses, 15 were focused on HS with only one study exploring folliculitis decalvans. While HS is a burdensome disease that is often comorbid with IBD, evaluating other FSDs and their relation to the gut–skin axis is important. Future studies should aim to include more FSDs when evaluating the gut–skin axis to provide a broader understanding of the microbiome’s impact.

Another limitation of this study includes the lack of data supporting prebiotic and probiotic therapies. Therapeutics supplementing probiotics have been previously studied among other dermatologic conditions including psoriasis or atopic dermatitis; however, there remains a lack of understanding of the utility of probiotic and prebiotic supplements for HS [9].

Finally, while many of the studies included in this review highlight the association of dysbiosis in HS and IBD, it is unclear whether the dysbiosis is involved in the pathogenesis of HS/IBD or if it is merely associated with one or both conditions. For example, clinical improvement was associated with a disappearance of *S. aureus* [25]. However, it is uncertain whether the disappearance of *S. aureus* is causing an improvement in symptoms or if it is a by-product of another pathogenic mechanism. Similarly, the impact of dysbiosis on morbidity is unclear. Longitudinal studies and additional clinical trials are necessary to establish causality and understand the efficacy of microbiome-targeted therapies and dietary interventions. Integrating microbiome analysis into routine clinical practice in patients with follicular disorders may eventually help guide a more personalized medicine approach that optimizes treatment.

## 5. Conclusions

In conclusion, this systematic review highlights the complex interplay of the microbiome in the pathogenesis of FSDs, particularly HS, and its association with IBD. Dietary modifications, routine microbiota analysis, and targeted immune modulation are promising approaches to managing these complex autoinflammatory conditions. However, more research evaluating the microbiome’s true impact on FSDs with comorbid IBD is needed.

## Figures and Tables

**Table 1:** Studies included for analysis and their characteristics, including patient population, sample size, and the follicular disorder studied. Abbreviations: HS; hidradenitis suppurativa, IBD; inflammatory bowel disease.

Author	Sample Size	Patient N, Control N	Follicular Disorder	IBD Included?	Gut/Skin Microbiome
Aboud et al. [15]	185	185, 0	HS	Yes	Gut
Cronin et al. [16]	252	157, 95	HS	Yes	Gut
Cannistra et al. [17]	12	12, 0	HS	No	Gut
Colboc et al. [18]	20	20, 0	HS	No	Gut
Assan et al. [19]	469	307, 162	HS	No	Gut
Eppinga et al. [20]	123	107, 33	HS	Yes	Gut
McCarthy et al. [21]	59	59, 50	HS	Yes	Skin and Gut
Nikolakis et al. [22]	50	50, 0	HS	No	Skin
Ogut et al. [23]	30	15, 15	HS	No	Gut
Guenin-Mace et al. [24]	66	32, 34	HS	No	Skin
Matard et al. [25]	40	20, 20	Folliculitis Decalvans	No	Skin
Riverain-Gillet et al. [26]	60	60, 17	HS	No	Skin
Lam et al. [10]	37	17, 20	HS	No	Gut
Hsu et al. [27]	34	22, 12	HS	No	Skin
Giudici et al. [3]	3	3, 0	HS	Yes	NR
Marzano et al. [28]	5	5, 0	HS	Yes	Skin

**Table 2:** Studies included for analysis, stratified by topic, evaluating follicular skin disorders and the microbiome. Quality assessments were conducted using the Newcastle–Ottawa Scale for Cohort Studies.

Category	Author, Year	Quality Assessment
Diet Analysis	Aboud, 2020 [15]	Good
	Cannistrà, 2013 [17]	Fair
	Colboc, 2016 [18]	Good
	Assan, 2020 [19]	Good
Colonization of Skin/Gut Microbiome	Eppinga, 2016 [20]	Good
	McCarthy, 2022 [21]	Good
	Nikolakis, 2017 [22]	Good
	Ogut, 2017 [23]	Good
	Guenin-Macé, 2020 [24]	Good
	Matard, 2020 [25]	Good
	Riverain-Gillet, 2020 [26]	Good
	Lam, 2021 [10]	Good
	Cronin, 2023 [16]	Good
	Hsu, 2022 [27]	Fair
Immunological/Serological Biomarkers	Giudici, 2015 [3]	Poor
	Marzano, 2014 [28]	Fair

**Table 3:** Studied microbiota, their compositional changes among included studies, and potential mechanisms of action. Abbreviations: HS; hidradenitis suppurativa, CD; Crohn’s disease.

Species	Microbiota Compositional Changes	Possible Mechanisms
*Enterococcus faecalis*	Present in gut in both HS and CD [20]	Secreting metalloproteases that degrade intestinal mucosa [29]
*Veillonella parvula*	Present in gut in both HS and CD [16]	Linked with higher nitrate levels, an inflammatory compound, promoting colonization [30]
*Escherichia-shigella*	Present in gut in both HS and CD, increased in contaminant HS and IBD patients [16,20]	Produces outer membrane vesicles to increase cytokine production [31]
*Ruminococcus gnavus*	Elevated in patients with HS and CD [21]	Synthesizes glucorhamnan, which induces TNFa secretion by dendritic cells [32]
*Staphylococcus aureus*	Found on lesioned skin among patients with folliculitis decalvans [25] Found in skinfolds in HS [28]	Acts as a potential reservoir for other abnormal flora [25]
*Prevotella* spp.*, Peptoniphilus* spp.	Found to be dominant at HS lesion sites [24,27]	Dysregulation of tryptophan catabolism [24]
*Robinsoniella*	Present in HS patients but not controls [10]	Potential invader of gut [10]
*Sellimonas*	Present in HS patients but not controls [10]	Has been linked to other chronic autoinflammatory conditions, etiology unknown [10]
*Actinomyces*	Abundant in HS skinfolds [24]	Dysregulation of tryptophan catabolism [24]
*Campylobacter ureolyticus*	Abundant in HS skinfolds [24]	Dysregulation of tryptophan catabolism [24]
*Mobiluncus*	Abundant in HS skinfolds [24]	Dysregulation of tryptophan catabolism [24]
*Faecalibacterium*	Depletion in HS patients and greater depletion in contaminant HS and IBD patients [16,20]	May be protective—secrete anti-inflammatory proteins that inhibit NF-kB in intestinal epithelial cells [33]

## Supplementary Materials

The following are available online at https://www.mdpi.com/article/10.3390/ijms251810203/s1.
