# Supplementary material for: Follicular Skin Disorders, Inflammatory Bowel Disease, and the Microbiome: A Systematic Review
Source: Int J Mol Sci. 2024 Sep 23;25(18):10203. doi: 10.3390/ijms251810203 (PMC11431962; doi:10.3390/ijms251810203)
Supplement: Supplementary file 1 [file ijms-25-10203-s001.zip › ijms-3186328-supplementary.pdf]

SEARCH STRATEGY

Hidradenitis suppurativa, Acne inversa, Pilonidal sinus, Acne conglobata, Dissecting cellulitis (of the scalp), Folliculitis decalvans, Perifolliculitis capitis abscedens et suffodiens, Sinus tracts, Inflammatory nodules
AND
Inflammatory bowel disease, Cohn's disease, Ulcerative colitis, Indeterminate colitis, Gut dysbiosis, Microbiome alteration, Microbiome, Gastrointestinal, Oral microbiome, Oral

Database	Search string	Date	Results
PubMed	("Hidradenitis Suppurativa"[Mesh] OR "Acne inversa"[tiab] OR "pilonidal sinus"[tiab] OR "Acne conglobata"[tiab] OR "dissecting cellulitis"[tiab] OR "folliculitis decalvans"[tiab] OR "perifolliculitis capitis abscedens et suffodiens"[tiab] OR "sinus tracts"[tiab] OR "inflammatory nodules"[tiab] OR "hidradenitis suppurativa"[tiab] OR HS[tiab] OR "gut-skin axis"[tiab] OR "suppurative hidradenitis"[tiab] OR "pilonidal abscess"[tiab] OR "pilonidal fistula"[tiab] OR "pilonidal cyst"[tiab] OR "sacrococcygeal sinus"[tiab] OR "sinus pilonidal"[tiab]) AND ("Inflammatory Bowel Diseases"[Mesh] OR "Colitis, Ulcerative"[Mesh] OR "Crohn Disease"[Mesh] OR "Crohn's disease"[tiab] OR "ulcerative colitis"[tiab] OR "Indeterminate **colitis"[tiab] OR "gut dysbiosis"[tiab] OR "microbiome alteration"[tiab] OR "Dysbiosis"[Mesh] OR dysbiosis[tiab] OR "inflammatory bowel disease*"[tiab] OR IBD[tiab])**	3/21/2024	683
Embase	('suppurative hidradenitis'/exp OR 'acne inversa' OR 'apocrinitis' OR 'hidradenitis suppurativa' OR 'suppurativa, hidradenitis' OR 'suppurative hidradenitis' OR 'pilonidal sinus'/exp OR 'barber hair sinus' OR 'coccygeal sinus' OR 'hair bearing sinus' OR 'pilonidal abscess' OR 'pilonidal cyst' OR 'pilonidal disease' OR 'pilonidal fistula' OR 'pilonidal sinus' OR 'pylonidal cyst' OR 'sacrococcygeal pilonidal cyst' OR 'sacrococcygeal pilonidal sinus' OR 'sacrococcygeal sinus' OR 'sinus pilonidalis' OR 'sinus pilonidalus' OR 'sinus, pilonidal' OR 'acne conglobata'/exp OR 'acne conglobata' OR 'perifolliculitis conglumerata suppurativa' OR 'folliculitis decalvans'/exp OR 'dissecting cellulitis'/exp OR 'dissecting cellulitis of the scalp'/exp OR 'hoffman`s abscessed and dissecting perifolliculitis' OR 'hoffman`s dissecting cellulitis' OR 'hoffman`s perifolliculitis' OR 'abscessive dissecting head perifolliculitis' OR 'dissecting cellulitis of scalp' OR 'dissecting cellulitis of the scalp' OR 'dissecting folliculitis' OR 'folliculitis abscedens et suffodiens' OR 'perifolliculitis abscedens et suffodien' OR 'perifolliculitis abscedens et suffodiens' OR 'perifolliculitis capitis abscedens' OR 'perifolliculitis capitis abscedens et suffodiens' OR 'scalp dissecting cellulitis' OR 'sinus tracts':ti,ab OR 'inflammatory nodules':ti,ab OR hs:ti,ab OR 'gut skin axis'/exp) AND ('inflammatory bowel disease'/exp OR 'inflammatory bowel disease' OR 'inflammatory bowel diseases' OR 'ulcerative colitis'/exp OR 'chronic ulcerative colitis' OR 'colitis ulcerativa' OR 'colitis ulcerosa' OR 'colitis ulcerosa chronica' OR 'colitis, mucosal' OR 'colitis, ulcerative' OR 'colitis, ulcerous' OR 'colon, chronic ulceration' OR 'histiocytic ulcerative colitis' OR 'mucosal colitis' OR 'ulcerative colitis' OR 'ulcerative colorectitis' OR 'ulcerative procto colitis' OR 'ulcerative proctocolitis' OR 'ulcerous colitis' OR 'crohn disease'/exp OR 'crohn disease' OR 'crohn`s disease' OR 'crohns disease' OR 'cleron disease' OR 'enteritis regionalis' OR 'intestinal tract, regional enteritis' OR 'morbus crohn' OR 'regional enteritis' OR 'regional enterocolitis' OR 'indeterminate colitis'/exp OR 'microbiome alteration':ti,ab OR 'dysbiosis'/exp OR 'dysbacteriosis' OR 'dysbiosis' OR ibd:ti,ab OR 'gut dysbiosis'/exp)	3/20/2024	1743

Web of Science	("Hidradenitis Suppurativa" OR "Acne inversa" OR "pilonidal sinus" OR "Acne conglobata" OR "dissecting cellulitis" OR "folliculitis decalvans" OR "perifolliculitis capitis abscedens et suffodiens" OR "sinus tracts" OR "inflammatory nodules" OR "hidradenitis suppurativa" OR HS OR "gut-skin axis" OR	3/20/2024	746

	"suppurative hidradenitis" OR "pilonidal abscess" OR "pilonidal fistula" OR "pilonidal cyst" OR "sacrococcygeal sinus" OR "sinus pilonidal")Topic<br>AND<br>("Inflammatory Bowel Diseases" OR "Colitis, Ulcerative" OR "Crohn Disease" OR "Crohn's disease" OR "ulcerative colitis" OR "Indeterminate colitis" OR "gut dysbiosis" OR "microbiome alteration" OR "Dysbiosis" OR dysbiosis OR "inflammatory bowel disease*" OR IBD)Topic		
Google Scholar	("Hidradenitis Suppurativa" OR "Acne inversa" OR "pilonidal sinus" OR "Acne conglobata" OR "dissecting cellulitis" OR "folliculitis decalvans" OR "perifolliculitis capitis abscedens et suffodiens" OR "sinus tracts" OR "inflammatory nodules" OR "hidradenitis suppurativa" OR HS OR "gut-skin axis" OR "suppurative hidradenitis" OR "pilonidal abscess" OR "pilonidal fistula" OR "pilonidal cyst" OR "sacrococcygeal sinus" OR "sinus pilonidal")<br>AND<br>("Inflammatory Bowel Diseases" OR "Colitis, Ulcerative" OR "Crohn Disease" OR "Crohn's disease" OR "ulcerative colitis" OR "Indeterminate colitis" OR "gut dysbiosis" OR "microbiome alteration" OR "Dysbiosis" OR dysbiosis OR "inflammatory bowel disease*" OR IBD)	3/21/2024	100 (first 10 pages when sorted by relevance)
Cochrane Central Register of Controlled Trials	ID<br>#1<br>all trees<br>Search Hits<br>MeSH descriptor: [Hidradenitis Suppurativa] explode 179<br>#2<br>conglobata" OR "dissecting cellulitis" OR "folliculitis decalvans" OR "perifolliculitis capitis abscedens et suffodiens" OR "sinus tracts" OR "inflammatory nodules" OR "hidradenitis suppurativa" OR "gut-skin axis" OR "suppurative hidradenitis" OR "pilonidal abscess" OR "pilonidal fistula" OR "pilonidal cyst" OR "sacrococcygeal sinus" OR "sinus pilonidal"<br>897<br>#3 MeSH descriptor: [Inflammatory Bowel Diseases] explode all trees 4804<br>● #4 (HS):ti,ab,kw 10398<br>● #5 MeSH descriptor: [Colitis, Ulcerative] explode all trees<br>"Acne inversa" OR "pilonidal sinus" OR "Acne	3/21/2024	224

2206

#6 MeSH descriptor: [Crohn Disease] explode all trees 2252

#7 MeSH descriptor: [Dysbiosis] explode all trees 212

#8 "Crohn's disease" OR "ulcerative colitis" OR "Indeterminate colitis" OR "gut dysbiosis" OR "microbiome alteration" OR dysbiosis OR "inflammatory bowel disease" OR "inflammatory bowel diseases" OR IBD 14646

#9 #1 OR #2 OR #4 11024

#10 #3 OR #5 OR #6 OR #7 OR #8 14722

#11 #9 AND #10 233

Trials only – 224
